# Publisher’s Note

**DOI:** 10.1080/21655979.2024.2326365

**Published:** 2024-03-01

Due to a publisher error, a retraction notice was incorrectly issued on the article ‘Mechanism of isosorbide dinitrate combined with exercise training rehabilitation to mobilize endothelial progenitor cells in patients with coronary heart disease’, https://doi.org/10.1080/21655979.2021.2000258. We have now removed the retraction notice.

We apologise to the authors for this error.

